# Heparanase Overexpresses in Keratoconic Cornea and Tears Depending on the Pathologic Grade

**DOI:** 10.1155/2017/3502386

**Published:** 2017-12-12

**Authors:** Beatriz García, Olivia García-Suárez, Jesús Merayo-Lloves, Guilherme Ferrara, Ignacio Alcalde, Javier González, Carlos Lisa, Jose F. Alfonso, Fernando Vazquez, Luis M. Quirós

**Affiliations:** ^1^Instituto Universitario Fernández Vega, Universidad de Oviedo & Fundación de Investigación Oftalmológica, Oviedo, Spain; ^2^Department of Functional Biology, University of Oviedo, 33006 Oviedo, Spain; ^3^Department of Morphology and Cell Biology, University of Oviedo, 33006 Oviedo, Spain; ^4^Department of Organic and Inorganic Chemistry, University of Oviedo, 33006 Oviedo, Spain; ^5^Department of Microbiology, Hospital Universitario Central de Asturias, Oviedo, Spain

## Abstract

**Background:**

Keratoconus has classically been defined as a noninflammatory disorder, although recent studies show elevated levels of inflammatory markers suggesting that keratoconus could be, at least in part, an inflammatory condition. Heparanase upregulation has been described in multiple inflammatory disorders. In this article, we study the differential expression of heparanase in cornea and tears from keratoconus patients and healthy controls.

**Methods:**

A transcriptomic approach was used employing quantitative polymerase chain reaction to analyze the expression of heparanase and heparanase 2 in stromal and epithelial corneal cells. The protein expression was analyzed by immunohistochemistry in corneal sections. Enzymatic activity in tears was measured using [^3^H]-labeled heparan sulfate as substrate.

**Results:**

Heparanase transcription was detected in stromal and epithelial cells and appeared upregulated in keratoconus. Overexpression of the enzyme was also detected by immunohistochemistry. Corneal expression of heparanase 2 was detected in some cases. Heparanase catalytic activity was found in tears and displayed a positive correlation with the degree of keratoconus.

**Conclusions:**

Heparanase overexpresses in keratoconic corneas, possibly reinforcing the inflammatory condition of the pathology. The presence of heparanase activity in tears allows us to propose its use as a biomarker for the diagnosis of the disorder.

## 1. Introduction

Keratoconus is a corneal ectasia that results in the cornea taking on a conical shape, causing severe astigmatism, scarring, and, for one in five patients, ultimately loss of vision and the need for corneal transplants [1].

Histologically, keratoconus displays many abnormal features which affect different layers of the cornea, including abnormal epithelial and stromal keratocyte shape, local thickening of the epithelium, Bowman's layer breakage, and thinning of the stroma [2, 3]. Keratoconus is likely a multifactorial, multigenetic disorder with complex inheritance patterns, and environmental factors probably play an equally important role in disease causation [4]. Although keratoconus has traditionally been viewed as a noninflammatory disease, reports of the presence of certain inflammatory mediators in keratoconus patients has led some authors to suggest that inflammation plays a role in the onset or progression of the pathology [5].

Proteoglycans (PGs) are a diverse group of glycoconjugates composed of various core proteins posttranslationally modified with linear, anionic polysaccharides called glycosaminoglycans (GAGs), consisting of repeating disaccharides. Heparan sulfate proteoglycans (HSPGs) comprise a reduced and specific group of proteins covalently linked to heparan sulfate (HS) GAG chains. HS is a complex biopolymer initially created as a chain of alternating D-glucuronic acid and N-acetylated-D-glucosamine. At various positions, the molecule is modified by a series of interdependent enzymatic reactions that include N-deacetylation of N-acetylated-D-glucosamine, usually followed by N-sulfation, epimerization of D-glucuronic acid into iduronate, and the addition of sulfate groups at C2 of uronic acid, and at C6 and C3 of glucosamine residues [6]. Chain modification results in clusters of highly sulfated and iduronate-rich regions separated by more flexible low or nonsulfated regions [7]. Specific sets of variably modified disaccharides, usually within the sulfated domains, can define binding sites for a multitude of specific ligands, including cytokines, chemokines, growth factors, enzymes and enzyme inhibitors, and extracellular matrix (ECM) proteins [6]. A variety of normal and pathological functions have been ascribed to HSPGs, including cell adhesion and migration, organization of the ECM, regulation of proliferation, differentiation and morphogenesis, cytoskeleton organization, tissue repair, inflammation, vascularization, and cancer metastasis [6, 8]. All corneal cells express HSPGs, which are ubiquitously present in tissues and are mainly associated with the cell surface and the ECM [6, 8]. Various eye diseases appear related to changes in PGs and GAGs, although there are few studies examining alterations in these molecules in connection with keratoconus [9–12]. Recently, however, there have been reports of changes in corneal stromal cells in keratoconus that point to an increase in HS chains and their levels of sulfation [13].

Heparanase (HPSE) is an endo-*β*-D-glucuronidase that cleaves specific linkages in the structure of the HS, yielding fragments which are able to contain biological activity. With a few exceptions, in normal noncancerous cells, the HPSE gene is not transcribed [14], although its expression has been reported in murine corneal epithelium and several retinal layers [15]. HPSE expression is induced in all major types of human cancer, is often associated with reduced patient survival and increased tumor metastasis [16], and has been shown to be associated with numerous inflammatory conditions, such as inflammatory bowel disease and rheumatoid arthritis [14]. Increased corneal expression of this molecule has also been reported during infection, most likely due to HPSE-positive infiltrating cells. [15]. Heparanase 2 (HPSE2) is a homologue of HPSE that lacks HS-degrading activity, although it is able to interact with HS with high affinity [16]. HPSE2 is capable of associating with HPSE, thereby possibly modulating its enzymatic activity and signaling properties [16, 17].

In this paper, we investigate the expression patterns of heparanase genes in the keratoconus cornea in comparison to healthy controls. The study analyzes both the transcription and the protein levels in the corneal tissues using qRT-PCR and immunohistochemistry. Taking into account that the tear proteome displays a highly dynamic character, we investigated the levels of heparanase enzymatic activity in tears of keratoconus patients of different grades in an effort to define its use as a biomarker for this eye disorder.

## 2. Methods

### 2.1. Materials

The following materials were purchased from the manufacturers indicated: RNeasy Kit and RNase-Free DNase from Qiagen (Hilden, Germany); High-Capacity cDNA Reverse Transcription Kit and Power SYBR Green PCR Master Mix from Applied Biosystems (Foster City, CA); GenElute PCR clean-up kit, 3-3′-diaminobenzidine, heparinase I and III blend from *Flavobacterium heparinum*, and heparan sulfate from Sigma-Aldrich (St. Louis, MO); EnVision™ Flex/HRP and Envision FLEX target retrieval solution of high pH from Dako (Glostrup, Denmark); cellulose acetate filter paper with pore size of 0.22 *μ*m was purchased from Sartorius Stedim (Göttingen, Germany); tetracaine hydrochloride and oxibuprocain hydrochloride were from Alcon Cusí (Barcelona, Spain); DMEM + F12 culture medium containing nonessential amino acids, RPMI 1640 Vitamin Solution 100x, 1% antibiotics (penicillin/streptomycin), and fetal bovine serum from Gibco (Waltham, MA); HiTrap Desalting column, superose 12, and [^3^H]acetic anhydride (500 mCi/mmol) from GE Healthcare Life Sciences (Little Chalfont, UK); Vivaspin 500 centrifugal filter units from Sartorius (Gotinga, Germany). All other chemicals were obtained from commercial sources and were of analytical grade.

The following antibodies were used in this study: goat anti-heparanase 1 polyclonal antibody (L-19) and rabbit polyclonal anti-heparanase 1 (H-80), both purchased from Santa Cruz Biotechnology Inc. (Santa Cruz, CA) and rabbit anti-heparanase-2 polyclonal antibody, purchased from Thermo Fischer Scientific (Waltham, MA). Anti-rabbit (sc-2004) and anti-goat (sc-2005) secondary antibodies were also from Santa Cruz Biotechnology (Santa Cruz, CA).

### 2.2. Isolation and Culture of Corneal Stromal Cells

Human central corneal tissue was obtained from cadaver donors and from penetrating keratoplasty interventions on patients suffering from keratoconus. Healthy donor tissues were screened and tested negative for HIV, hepatitis B and C virus, and syphilis and were not usable for human corneal transplantation.

The epithelium was removed with ethanol (70%, 30 s) and a spatula, and the endothelium by Descemet membrane endothelial keratoplasty, after which the absence of epithelial and endothelial cells was assessed by microscopy. Corneal stromal cells were obtained by digesting 2 mm diameter pieces from the central cornea in 0.25% trypsin/ethylenediaminetetraacetic acid solution for 30 min at 37°C. After centrifugation, the supernatant was discarded and the pellet resuspended in a DMEM + F12 culture medium containing nonessential amino acids, RPMI 1640 Vitamin Solution 100x, 1% antibiotics (penicillin/streptomycin), and 10% fetal bovine serum. When cultures reached 80% confluence, they were replated at a density of 2 × 10^5^ cells/ml in 75 cm^2^ polystyrene flasks and incubated at 37°C in a 5% (*v*/*v*) CO_2_ atmosphere. As a control to evaluate whether cells maintained a stable phenotype, we performed alpha-smooth muscle actin immunostaining; this staining remained negative at least until the fifth passage, suggesting that the stromal cells did not readily differentiate to myofibroblasts. As a result, only cultures in passages 1–5 were used. In all studies carried out in this work, the different cell lines were analyzed separately.

### 2.3. Obtaining Corneal Epithelial Samples by Impression Cytology

All the samples used in this study were obtained from patients (*n* = 8) from the Instituto Oftalmológico Fernández-Vega (IOFV, Asturias, Spain) and from healthy volunteers (*n* = 5). Informed oral and written consent of the patients and volunteers was obtained under a protocol approved by the Ethical Committee of the IOFV in accordance with the guidelines of the Tenets of the Declaration of Helsinki.

Prior to impression, one drop of local anaesthetic (0.1% tetracaine hydrochloride and 0.4% oxibuprocain hydrochloride) was instilled into the eye and excessive tear fluid and medication were wiped away. Next, a 6 mm disc of cellulose acetate paper was applied gently onto the corneal surface with the aid of sterile blunt forceps. The paper was allowed to remain in contact with the central cornea for 5 seconds, peeled off, turned over, and the other side applied for an additional period of 5 seconds. The paper was immediately transferred into RNase-free tubes and rapidly frozen and stored at −80°C.

### 2.4. Obtaining Tear Fluid Samples

Tear fluid was obtained from patients (*n* = 42) and healthy volunteers (*n* = 11) with the same range of ages by using a sterile capilar tube and applying gentle suction with a syringe. The fluid was immediately transferred into sterile tubes containing 20% of the final volume in glycerol and rapidly frozen and stored at −80°C. A minimum of 10 *μ*l of tear fluid was required to be included as a valid sample.

### 2.5. RNA Isolation and cDNA Synthesis

Tissue fragments (20 and 30 mg in weight) were homogenized using a polytron PT 2100 (Kinematica Inc.; Bohemia, NY), and RNA was isolated using the RNeasy kit (Qiagen, Hilden, Germany) and processed as previously described [18]. To ensure removal of residual contaminating DNA, samples were subjected to treatment with RNase-free DNase during the purification process itself. The concentration of RNA obtained was determined spectrophotometrically by measuring absorbance at 260 nm of a 1 : 50 dilution using a BioPhotometer (Eppendorf; Hamburg, Germany). cDNA synthesis was carried out using the High Capacity cDNA Transcription Kit (Applied Biosystems, Foster City, CA, USA). The reactions were performed using a thermocycler iCycler IQ (BioRad; Hercules, CA), using 2 *μ*g of RNA as starting material. The reaction products were cleaned using the PCR Clean-Up GenElute Kit in line with the manufacturer's instructions. Finally, the aliquots containing the cDNA were diluted 1 : 20 with water and used for qRT-PCR assays or stored at −20°C until use.

### 2.6. Quantitative Real-Time Polymerase Chain Reaction (qRT-PCR) and Data Analysis

The primer sequences were *HPSE* (gene ID 10855) forward 5′-ATGCTCAGTTGCTCCTGGAC-3′, reverse 5′-CTCCTAACTGCGACCCATTG-3′ and *HPSE2* (gene ID 60495) forward 5′-CACCCTGATGTTATGCTGGAG-3′, reverse 5′-TCCAGAGCAATCAGCAAAGTTA-3′. qRT-PCR reactions, and analysis of amplimer products were carried out accordingly to the methods already detailed [19]. Actin was included on each plate as a control gene to compare run variation and to normalize individual gene expression. Statistical analysis of the data and expression of the values of differential transcription were performed as previously described [18].

### 2.7. Immunohistochemistry

Tissue sections from the central cornea, prepared as previously described [13], were dewaxed, and the rehydrated sections were rinsed in phosphate-buffered saline containing 1% Tween-20, and then immunostained as previously described [13].

### 2.8. Radioisotopic Labeling and Molecular Size Fractionation of HS

HS was partially de-N-acetylated and re-N-acetylated with [^3^H]acetic anhydride according to the method previously described [20]. Molecular size fractionation of HS was carried out using size-exclusion chromatography. 20 *μ*Ci of [^3^H]-HS was applied to a superose 12 column connected to an FPLC system (GE) and eluted with 50 mM of pH 8.0 Tris-HCl buffer and 150 mM of NaCl at a flow rate of 1 ml/min. 1 ml fractions were collected, and HS eluting from the column was determined by measuring the radioactivity in 200 *μ*l aliquots of each fraction. Fractions including molecules of high molecular weight were pooled and precipitated with 85% ethanol for 2 h at −80°C. The identity of the polysaccharide was confirmed by exclusion chromatography in the same conditions after treatment with a mixture of heparinases I and III.

### 2.9. Heparanase Assay

The ability of the tears to fragment HS chains was monitored by ultrafiltration. The reactions were carried out in a total volume of 200 *μ*l that included 100 mM citrate buffer pH 5.3, 1 mM CaCl_2,_ 20 nCi of [^3^H]-HS and 10 *μ*l of tears. The reactions were incubated for 16 h at 37°C and stopped by the addition of 220 *μ*l of 2 M NaCl and 200 *μ*l of chloroform. After vigorous shaking, the sample was centrifuged at 10,000 ×g and 200 *μ*l of the aqueous phase was extracted. 100 *μ*l of extract was ultrafiltered using filters with an exclusion size of 5 kDa which had previously been treated with 500 ml of 20% glycerol. Filtration was carried out at 12,000 ×g for 5 minutes, and the reaction was measured by determining the radioactivity in aliquots of the filtrate.

### 2.10. Statistical Analysis

All analyses were performed using the Statistics for Windows program (Statsoft Inc.; Tulsa, OK). Mean values were compared between two samples by the Mann–Whitney *U* test and between multiple samples by the Kruskal–Wallis test. Correlations were assessed by Pearson's correlation coefficient. *p* < 0.05 was accepted as significant.

## 3. Results

**Figure 1:**
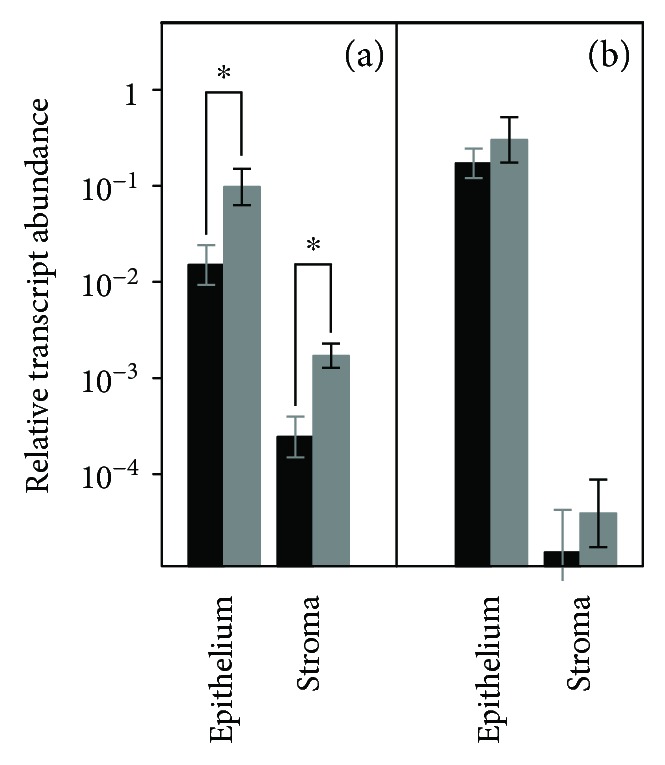
Differential transcription of genes encoding heparanases. Relative abundance for healthy cells (black bars) and keratoconic cells (gray bars) are plotted for HPSE (a) and HPSE2 (b). Values on the *y*-axis are on a logarithmic scale. ^∗^*p* < 0.001; error bars, standard deviations.

Analysis of the differential transcription of the genes encoding heparanases in keratoconic corneas was carried out by qRT-PCR. The study was conducted independently for corneal stroma (using cultures of different stromal cells lines obtained from different healthy corneas and keratoconic patients) and for epithelium (using samples obtained by impression cytology). In healthy cornea, HPSE transcripts could be detected both in the stromal and in the epithelial cells, although levels were about two orders of magnitude higher in the latter. Keratoconic cornea also displayed HPSE transcripts in both types of tissue, although it is worth noting that it was overexpressed, about 6- to 7-fold, compared to the respective values observed in healthy individuals (Figure 1), differences which were statistically significant (*p* < 0.001). HPSE2 transcripts were also detected, mainly in epithelial cells, although not in all the individuals analyzed, and the values found in healthy tissue did not display significant differences from keratoconic cornea (Figure 1).

**Figure 2:**
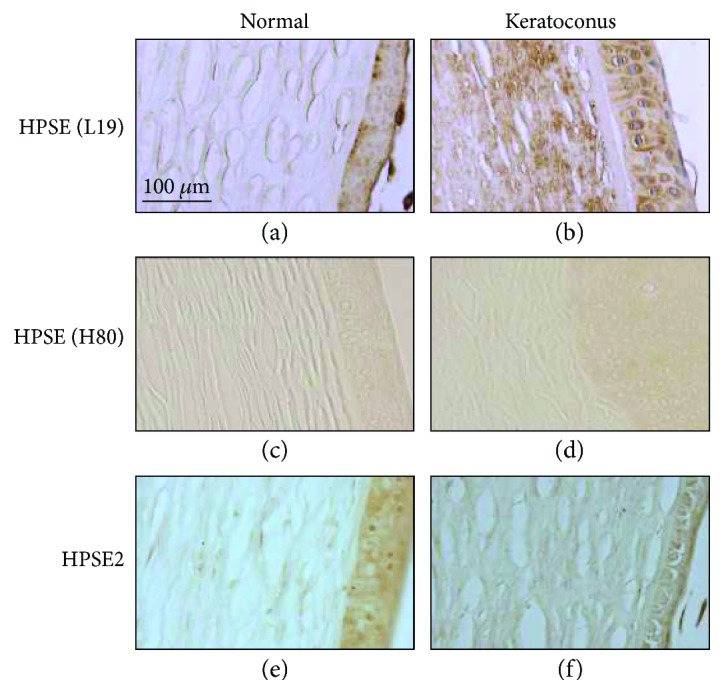
Immunohistochemistry of heparanases. (A–D) Immunohistochemistry of HPSE carried out using the antibody L-19 (A, B) and the antibody H-80 (C, D). (E, F) Immunohistochemistry of HPSE2. The column on the left shows the results obtained using healthy corneal sections, and that on the right the results using keratoconic corneal sections.

HPSE is synthesized as a proenzyme of 61.2 kDa, which is cleaved by cathepsin L to generate the active form consisting of 8 and 50 kDa subunits that associate noncovalently [16]. We evaluated changes in the expression of the HPSE protein by immunohistochemistry using two different antibodies. L-19 antibody recognizes peptide mapping near the C-terminus of the molecule and is consequently present in both the catalytically active form and in the latent precursor. Using this antibody on healthy corneas resulted in certain levels of staining, predominantly in the basal layers of the epithelium (Figure 2, A). In contrast, the corneas of keratoconus patients showed significant increases in staining, both in the epithelium and in the stroma; at the level of the epithelium, a homogeneous overexpression in keratoconus was observed, while the stroma showed a particularly intense overexpression, predominantly in the subepithelial region (Figure 2, B). H-80 antibody is produced against amino acids 101–180 so that it is able to recognize only the inactive precursor. Immunohistochemical analysis of corneas using this antibody resulted in faint stainings, mainly detectable in keratoconic epithelia (Figure 2, C and D). Furthermore, immunostaining of corneas with anti-heparanase 2 antibodies allowed the detection of certain levels of expression, especially in the epithelium, suggesting the presence of the protein in these corneas (Figure 2, E and F).

**Figure 3:**
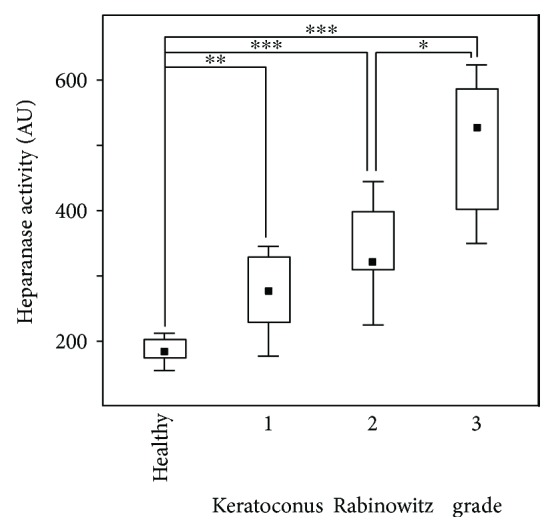
Heparanase activity in tears from healthy individuals and patients with different grades of keratoconus. The hydrolysis of [^3^H]-HS molecules into fragments capable of passing through 5 kDa filters is shown in arbitrary units. The rectangles represent 25%–75% ranges, and the squares inside them the median value. Statistically significant differences are indicated: ^∗^*p* < 0.05; ^∗∗^*p* < 0.01; ^∗∗∗^*p* < 0.001.

The presence of endo-*β*-D-glucuronidase activity in tears was tested for by determining the appearance of ultrafilterable HS fragments of low molecular weight resulting from the hydrolysis of high molecular weight [^3^H]-labeled HS. The results showed that low levels of HPSE activity could be detected in tears of healthy individuals and that in keratoconic individuals the level progressively increased as the Rabinowitz grade of the keratoconus increased (Figure 3). Differences between tears from keratoconic and healthy individuals were statistically significant in all the cases, as they were between the different grades except for between grades 1 and 2. This case, though, approached significance (*p* = 0.07), suggesting that it may well be positive if a wider sample of patients were used. The data showed a strong positive correlation between HPSE activity and the grade of the keratoconus (*r* = 0.89, *p* < 0.001).

## 4. Discussion

HSPGs are present in all types of human cells, albeit that HS species from different sources differ in terms of molecular size and their overall patterns of chain modification. Based on their structural diversity, HS chains are able to selectively interact with many different types of soluble and insoluble proteins, lipids, and even microorganisms, thereby modulating numerous cellular activities, including cell adhesion and migration, organization of the ECM, regulation of proliferation, differentiation and morphogenesis, cytoskeleton organization, tissue repair, inflammation, vascularization, and cancer metastasis [6, 8]. Given the importance of the biological functions in which it participates, the enzymatic remodeling of HSPGs profoundly affects a wide variety of physiological and pathological processes [14, 16]. HPSE is the sole human endoglycosidase that cleaves HS, although this molecule also displays various nonenzymatic activities [16, 17].

Whereas HS is produced by virtually all cells in the body, HPSE expression is kept tightly regulated at the transcriptional and posttranslational levels, since any uncontrolled cleavage of HS could result in significant tissue damage [14, 16]. HPSE expression in noncancerous cells has been reported to be restricted to certain specific cases, such as in the placenta, activated immune cells, and keratinocytes [21]. With respect to its expression in ocular tissues, studies in murine eyes have reported constitutive expression in the corneal epithelium and several retinal layers [15]. In this article, we report the expression of certain levels of HPSE in healthy corneas, predominantly in the basal layers of the epithelium. It is of note that corneas of keratoconus patients significantly overexpressed HPSE at both the epithelial and stromal level. Upregulation of HPSE associated with pathological processes has been widely described, including tumors, inflammatory bowel disease, rheumatoid arthritis, diabetic nephropathy, or atherosclerosis [14, 16, 22]. Increased levels of HPSE have also been related to ocular pathologies, such as the overexpression in corneal epithelium and stroma during infection with *Pseudomonas aeruginosa* [15] and in the vitreous of patients with proliferative diabetic retinopathy [23].

The upregulation of HPSE in cornea associated with *Pseudomonas* infection has been related to HPSE-positive infiltrating cells, and it was not able to be detected in corneas from immunized mice since they had a lower inflammatory response [15]. However, in our study, we were able to detect an approximately 6- to 7-fold increase in the transcription of the gene using epithelial cells, obtained by impression cytology, and cultured stromal cells, clearly suggesting that the observed overexpression occurs as a result of increased transcription in the corneal cells themselves. The protein is first synthesized as a latent 65 kDa proenzyme that is secreted via vesicles that bud from the Golgi apparatus, which then interacts with cell membrane HSPGs and other receptors, accumulates in endosomes, and undergoes processing at two proteolytic cleavage sites, located at Glu109-Ser110 and Gln157-Lys158, yielding 8- and 50-kDa subunits that heterodimerize to form the active enzyme. Using an antibody (H80) that recognizes an internal region present only in the 65 kDa form of the protein, we were able to detect immunostaining in epithelial cells, particularly in keratoconic epithelial cells. These immunostainings showed faint labeling, as would be expected for a molecular species that is temporary, in contrast to the intense labeling obtained for the final, processed HPSE. Together, these results indicate that the transcriptional and posttranslational regulation of the expression of the molecule occurs in the corneal cells.

HPSE upregulation, locally expressed at the site of inflammation, has been shown in multiple organ systems as well as in several autoimmune and human autoinflammatory disorders, although its precise mode of action is not completely understood [14]. The enzymatic degradation of HS affects several aspects of inflammatory response, including the release of cytokines and chemokines, the activation of immune cells, and leukocyte recruitment [14]. Keratoconus has traditionally been defined as a noninflammatory disorder due to the lack of neovascularization and cellular infiltration [24]. However, recent studies have shown the significant role of proteolytic enzymes, cytokines, and free radicals. Moreover, evidence increasingly supports the notion that thinning and ectasia of the cornea are related to a degraded extracellular matrix involving inflammatory events, which include increased levels of matrix metalloproteinases [24] and which might be reinforced by the action of HPSE. It has also been described that loss of corneal epithelial sulfate leads to corneal degeneration [25], and the health of the ocular surface involves soluble factors whose action may be strongly influenced by HPSE, as in the case of lacritin [26]. Some authors have proposed that the definition of inflammation should not necessarily be limited to the absence of neovascularization and lack of marked cellular infiltration [25]. Taking this line, the upregulation of HPSE in keratoconus could be added to the elevated levels of other inflammatory markers to suggest that keratoconus could be, at least in part, an inflammatory condition.

HPSE2 is a homologue of HPSE that lacks HS-degrading activity, although it is able to interact with HS with high affinity and is capable of modulating HPSE enzymatic activity and signaling properties, such that an antimetastatic character has been proposed for it [16]. Although it has previously been suggested that this molecule is not expressed in the eye [27], in the current work, it was indeed possible to detect transcripts in epithelial cells, although not in all the cases analyzed. Immunohistochemistry also detected certain levels of protein, although no significant differences were found between normal and keratoconic cells.

Mature HPSE is located in lysosomes, which are not considered typical secretory vesicles. Nevertheless, they may secrete their content in response to local or systemic cues, which releases the enzymatically active molecule and other molecules like cathepsins into the extracellular milieu [16, 28]. It has been reported that HPSE secretion increases in response to proinflammatory cytokines such as TNF*α*, although the effective stimuli vary among cell types and biological settings [28]. Altered levels of inflammatory cytokines, including TNF*α*, have been reported in tears of keratoconic patients, as well as elevated levels of cathepsins [5, 29]. Using [^3^H]-labeled HS, we were able to detect the presence of certain low levels of HPSE catalytic activity in tears from healthy individuals. This activity greatly increased in tears of keratoconic patients, and the values showed a strong positive correlation with the grade of keratoconus. The tear proteome displays a highly dynamic character that may reflect the altered states of specific eye disorders, as has been described in meibonian gland disease [30], autoimmune thyroid eye disease [31], pterygium [32], ocular rosacea [33], blepharitis [34], diabetes [35], and dry eye [36]. In the case of keratoconus, previous reports have shown differences in the tear protein profile [37], although the changes found do not include HPSE, probably because the methodology used does not directly detect the protein, but rather its catalytic activity, which allows higher sensitivity.

## 5. Conclusions

In summary, this work describes an overexpression of HPSE in keratoconic corneas that affects both the epithelium and the stroma. The presence of catalytic activity in tears is also reported, and this activity shows a positive correlation with the grade of keratoconus, thus allowing its use as a biomarker for the diagnosis of the disorder.

## References

[1] Romero-Jiménez M., Santodomingo-Rubido J., Wolffsohn J. S. (2010). Keratoconus: a review. *Contact Lens & Anterior Eye*.

[2] Zhang Y., Mao X., Schwend T., Littlechild S., Conrad G. W. (2013). Resistance of corneal RFUVA–cross-linked collagens and small leucine-rich proteoglycans to degradation by matrix metalloproteinases. *Investigative Ophthalmology & Visual Science*.

[3] Khaled M. L., Helwa I., Drewry M., Seremwe M., Estes A., Liu Y. (2017). Molecular and histopathological changes associated with keratoconus. *Biomed Research International*.

[4] Vazirani J., Basu S. (2013). Keratoconus: current perspectives. *Clinical Ophthalmology*.

[5] Jun A. S., Cope L., Speck C. (2011). Subnormal cytokine profile in the tear fluid of keratoconus patients. *PLoS One*.

[6] Sarrazin S., Lamanna W. C., Esko J. D. (2011). Heparan sulfate proteoglycans. *Cold Spring Harbor Perspectives in Biology*.

[7] Mobli M., Nilsson M., Almond A. (2008). The structural plasticity of heparan sulfate NA-domains and hence their role in mediating multivalent interactions is confirmed by high-accuracy ^15^N-NMR relaxation studies. *Glycoconjugate Journal*.

[8] Lindahl U., Kjellén L. (2013). Pathophysiology of heparan sulphate: many diseases, few drugs. *Journal of Internal Medicine*.

[9] Funderburgh J. L., Funderburgh M. L., Rodrigues M. M., Krachmer J. H., Conrad G. W. (1990). Altered antigenicity of keratan sulfate proteoglycan in selected corneal diseases. *Investigative Ophthalmology & Visual Science*.

[10] Funderburgh J. L., Hevelone N. D., Roth M. R. (1998). Decorin and biglycan of normal and pathologic human corneas. *Investigative Ophthalmology & Visual Science*.

[11] Akhtar S., Bron A. J., Hayes A. J., Meek K. M., Caterson B. (2011). Role of keratan sulphate (sulphated poly-*N*-acetyllactosamine repeats) in keratoconic cornea, histochemical, and ultrastructural analysis. *Graefe's Archive for Clinical and Experimental Ophthalmology*.

[12] Chaerkady R., Shao H., Scott S. G., Pandey A., Jun A. S., Chakravarti S. (2013). The keratoconus corneal proteome: loss of epithelial integrity and stromal degeneration. *Journal of Proteomics*.

[13] García B., García-Suárez O., Merayo-Lloves J. (2016). Differential expression of proteoglycans by corneal stromal cells in keratoconus. *Investigative Ophthalmology & Visual Science*.

[14] Meirovitz A., Goldberg R., Binder A., Rubinstein A. M., Hermano E., Elkin M. (2013). Heparanase in inflammation and inflammation-associated cancer. *FEBS Journal*.

[15] Berk R. S., Dong Z., Alousi S., Kosir M. A., Wang Y., Vlodavsky I. (2004). Murine ocular heparanase expression before and during infection with *Pseudomonas aeruginosa*. *Investigative Ophthalmology & Visual Science*.

[16] Arvatz G., Shafat I., Levy-Adam F., Ilan N., Vlodavsky I. (2011). The heparanase system and tumor metastasis: is heparanase the seed and soil?. *Cancer and Metastasis Reviews*.

[17] Levy-Adam F., Feld S., Cohen-Kaplan V. (2010). Heparanase 2 interacts with heparan sulfate with high affinity and inhibits heparanase activity. *Journal of Biological Chemistry*.

[18] Fernández-Vega I., García O., Crespo A. (2013). Specific genes involved in synthesis and editing of heparan sulfate proteoglycans show altered expression patterns in breast cancer. *BMC cancer*.

[19] García B., García-Suárez O., Fernández-Vega I., Vallina A., Astudillo A., Quirós L. M. (2016). Heparanase and heparanase 2 display differently deregulation in neuroendocrine tumors, depending on their differentiation grade. *Histology and Histopathology*.

[20] Freeman C., Parish C. R. (1997). A rapid quantitative assay for the detection of mammalian heparanase activity. *Biochemical Journal*.

[21] Hulett M. D., Freeman C., Hamdorf B. J., Baker R. T., Harris M. J., Parish C. R. (1999). Cloning of mammalian heparanase, an important enzyme in tumor invasion and metastasis. *Nature Medicine*.

[22] Vlodavsky I., Blich M., Li J. P., Sanderson R. D., Ilan N. (2013). Involvement of heparanase in atherosclerosis and other vessel wall pathologies. *Matrix Biology*.

[23] Abu El-Asrar A. M., Alam K., Nawaz M. I. (2015). Upregulated expression of heparanase in the vitreous of patients with proliferative diabetic retinopathy originates from activated endothelial cells and leukocytes. *Investigative Ophthalmology & Visual Science*.

[24] Galvis V., Sherwin T., Tello A., Merayo J., Barrera R., Acera A. (2015). Keratoconus: an inflammatory disorder?. *Eye*.

[25] Coulson-Thomas V. J., Chang S. H., Yeh L. K. (2015). Loss of corneal epithelial heparan sulfate leads to corneal degeneration and impaired wound healing. *Investigative Ophthalmology & Visual Science*.

[26] McKown R. L., Wang N., Raab R. W. (2009). Lacritin and other new proteins of the lacrimal functional unit. *Experimental Eye Research*.

[27] Zhang Y., Ryan D. S., Bower K. S., Ilan N., Vlodavsky I., Laurie G. W. (2010). Focus on molecules: heparanase. *Experimental Eye Research*.

[28] Ilan N., Elkin M., Vlodavsky I. (2006). Regulation, function and clinical significance of heparanase in cancer metastasis and angiogenesis. *The International Journal of Biochemistry & Cell Biology*.

[29] Balasubramanian S. A., Wasinger V. C., Pye D. C., Willcox M. D. (2013). Preliminary identification of differentially expressed tear proteins in keratoconus. *Molecular Vision*.

[30] Tsai P. S., Evans J. E., Green K. M. (2006). Proteomic analysis of human meibomian gland secretions. *British Journal of Ophthalmology*.

[31] Okrojek R., Grus F. H., Matheis N., Kahaly G. J. (2009). Proteomics in autoimmune thyroid eye disease. *Hormone and Metabolic Research*.

[32] Zhou L., Beuerman R. W., Ang L. P. (2009). Elevation of human α-defensins and S100 calcium-binding proteins A8 and A9 in tear fluid of patients with pterygium. *Investigative Ophthalmology & Visual Science*.

[33] Määttä M., Kari O., Tervahartiala T. (2006). Tear fluid levels of MMP-8 are elevated in ocular rosacea–treatment effect of oral doxycycline. *Graefe's Archive for Clinical and Experimental Ophthalmology*.

[34] Koo B. S., Lee D. Y., Ha H. S., Kim J. C., Kim C. W. (2005). Comparative analysis of the tear protein expression in blepharitis patients using two-dimensional electrophoresis. *Journal of Proteome Research*.

[35] Herber S., Grus F. H., Sabuncuo P., Augustin A. J. (2001). Changes in the tear protein patterns of diabetic patients using two-dimensional electrophoresis. *Advances in Experimental Medicine and Biology*.

[36] Grus F. H., Sabuncuo P., Herber S., Augustin A. J. (2002). Analysis of tear protein patterns for the diagnosis of dry eye. *Lacrimal Gland, Tear Film, and Dry Eye Syndromes 3*.

[37] Acera A., Vecino E., Rodríguez-Agirretxe I. (2011). Changes in tear protein profile in keratoconus disease. *Eye*.

